# Laparoscopic vs. robotic sacrocolpopexy: influence of age, BMI, and parity on perioperative outcomes

**DOI:** 10.3389/fsurg.2025.1625404

**Published:** 2025-08-29

**Authors:** Mila Strauss, Lieven N. Kennes, Janina Dombrowski, Elmar Stickeler, Charlotte Gräf, Katharina Lube, Alessia Hillmeyer, Laila Najjari

**Affiliations:** ^1^ Department of Obstetrics and Gynecology, University Hospital RWTH Aachen, Aachen, Germany; ^2^ Department of Economics and Business Administration, University of Applied Sciences Stralsund, Stralsund, Germany

**Keywords:** laparoscopic, obesity, pelvic organ prolapse, robotic, sacrocolpopexy

## Abstract

**Background:**

Laparoscopic and robotic-assisted sacrocolpopexy are established techniques for pelvic organ prolapse (POP) repair, but their performance in patients with higher age, BMI, and parity remains underexplored.

**Methods:**

In this retrospective single-center study, we analyzed 162 women undergoing minimally invasive sacrocolpopexy between 2010 and 2023: *n* = 104 via laparoscopic sacrocolpopexy (LSC) and *n* = 58 via robotic-assisted sacrocolpopexy (RASC). Patients were included if they had symptomatic or asymptomatic POP stage II or higher. Primary outcomes were surgical duration and length of postoperative hospital stay; secondary outcomes included intra- and postoperative complications. Regression analyses were used to assess the influence of age, BMI, and number of births.

**Results:**

Mean patient age was 64 ± 11.2 years. Surgical duration increased significantly with BMI (+2.82 min/unit, 95% CI: 0.50, 5.14, *p* = 0.0177) and parity (+9.8 min/birth, CI: 0.56, 19.14, *p* = 0.0379) in the LSC group, but not significantly in RASC (Surgical duration: (+2.00 min/unit, 95% CI: −0.53, 4.63, *p* = 0.1167; parity: + 8.7 min/birth, 95% CI: 0.50, 5.14, *p* = 0.0698). Postoperative stay was significantly prolonged with higher age (95% CI = 0.006, 0.057, *p* = 0.0152), BMI (95% CI = 0.019, 0.154, *p* = 0.0130), and number of vaginal births (95% CI = 0.008, 0.59, *p* = 0.01) in LSC, while these associations were attenuated in RASC (age: 95% CI: −0.0213, 0.0249, *p* = 0.876; BMI: −95% CI: −0.038, 0.060, *p* = 0.667; vaginal birth: 95% CI = 0.10, 0.44, *p* = 0.003). Overall complication rates exhibited no measurable difference between the groups (LSC 18%, RASC 19%). Complications were more frequent with fixation at the vaginal vault than the cervix.

**Conclusion:**

Robotic-assisted sacrocolpopexy appears to offer greater procedural consistency in patients with advanced age, obesity, and higher parity. These findings support the selective use of robotic assistance in anatomically or clinically complex cases and add to the limited evidence guiding personalized surgical planning in POP repair.

## Introduction

Pelvic organ prolapse (POP) is a common condition affecting up to 50% of women on clinical examination, though only 3%–6% are symptomatic (1). Risk factors such as advanced age, increasing parity, and obesity contribute significantly to its development. The condition predominantly affects postmenopausal women (2). With more than 60% of adults in OECD countries now classified as overweight or obese (3), these risk factors are increasingly relevant for clinical decision-making in POP management.

POP can be managed conservatively or surgically, depending on the severity of the condition and patient preferences. Conservative approaches include pelvic floor muscle training and pessary use, aiming to alleviate symptoms and improve quality of life. Surgical intervention is typically reserved for advanced cases or when conservative measures are ineffective (4, 5). Among surgical options, minimally invasive sacrocolpopexy—either laparoscopic (LSC) or robotic-assisted (RASC)—has become the gold standard due to reduced morbidity and quicker recovery compared to open surgery (6–8). Although LSC was first introduced in 1994, RASC gained traction following the introduction of the Da Vinci® system in 2005. RASC offers ergonomic and visual advantages, particularly in complex cases such as obesity (7, 9).

However, previous studies suggest comparable outcomes between both techniques, with some trade-offs in cost, learning curve, and operative time (9, 10). Notably, a recent study by Billone et al. (2024) compared robotic and mini-laparoscopic colposacropexy in a general population and found shorter operative times, reduced intraoperative blood loss, and less postoperative pain in the mini-laparoscopy group, despite similar hospital stays and complication rates. However, most existing studies evaluate these techniques in general populations and do not sufficiently address how patient-specific factors such as age, body mass index (BMI), and parity may influence surgical performance. This represents a critical knowledge gap, particularly as the global population continues to age and obesity rates rise. Given the ergonomic and technical advantages of robotic systems, it is plausible that RASC may offer measurable benefits over LSC in high-risk or anatomically challenging subgroups.

This retrospective study aims to address this gap by comparing surgical outcomes—specifically operative time, length of stay, and complication rates—between LSC and RASC across distinct patient demographics. By focusing on women with advanced age, elevated BMI, and higher parity, we seek to determine whether robotic assistance provides added clinical value in optimizing outcomes for these increasingly prevalent patient profiles.

## Patients and methods

### Study design

Given the retrospective design of the study, no formal power or sample size calculation was conducted. Instead, the study cohort is based on a complete enumeration of all eligible patients who presented to the Department of Urogynecology at University Hospital Aachen, Germany, between March 2010 and May 2023. Ethical approval was granted by the institutional ethics committee (Approval No. EK 085/11), and informed consent was waived due to the retrospective nature of the analysis.

No missing data were present in the dataset; therefore, no imputation procedures were required. Four patients were excluded from outcome analyses: two had mesh removals without reimplantation, one experienced an intraoperative bladder injury that led to procedure abortion, and one developed severe postoperative complication with a 35-day hospitalization, which was excluded from LOS analysis due to unrelated secondary issues.

### Eligibility criteria

A multidisciplinary team, including urogynecologists, specialized nurses, and colorectal surgeons, preoperatively assessed patients with pelvic organ prolapse (POP) based on a standardized internal protocol. Women diagnosed with POP stage II or higher (according to POP-Q classification) who underwent laparoscopic or robotic-assisted sacrocolpopexy with implantation of a polyvinylidene fluoride (PVDF) mesh were included.

The study population comprised both symptomatic women (e.g., those presenting with bulge symptoms, dragging sensations in the vagina, or bladder-, bowel-, and sexual dysfunction) as well as asymptomatic women. Asymptomatic patients were referred by private gynecologists and included based on objective findings during clinical and ultrasound examination. Their inclusion was justified by significant anatomical prolapse that, despite lacking symptoms, met surgical treatment thresholds. Patients were included for surgical treatment only if they had symptomatic POP stage II or higher; no patients with stage I prolapse underwent surgery. The surgical indication was based on both anatomical findings and the associated symptom burden, including vaginal pressure, pelvic pain, foreign body sensation, bladder voiding dysfunction, and incontinence (urge, stress, or mixed). In our study, we did not differentiate between grades II or higher, as these present with similar symptoms and identical therapeutic indications (11).

Patients were excluded if unfit for general anesthesia or unable to tolerate Trendelenburg position. Previous POP surgery was not considered an exclusion criterion. In clinical practice, recurrent prolapse is a frequent indication for surgery, and patients with prior POP procedures often require further interventions. Therefore, including such patients ensures that the study population reflects real-world clinical scenarios. Only patients with recurrent POP and concurrent symptomatic presentation were considered for surgery. The indication was based on clinically significant complaints such as vaginal bulge sensation, pelvic pain, bladder emptying disorder, urge or stress incontinence, or mixed incontinence. As a result, all patients in the study, including those with a history of previous POP surgery, presented with a clear indication for operative treatment.

### Interventions and comparator

All patients underwent either laparoscopic sacrocolpopexy (LSC) or robotic-assisted sacrocolpopexy (RASC). All procedures were performed by the same experienced urogynecologic surgeon, with over 10 years of experience in laparoscopic pelvic floor surgery and robotic-assisted procedures at the time of data collection.

Assignment to the surgical approach was not randomized and depended on patient characteristics, logistical factors (e.g., robotic system availability), and surgeon's preference. Both groups received PVDF mesh implantation using a standardized surgical technique.

### Surgical indications

The primary indication for surgery was apical prolapse, involving either the cervix or the vaginal vault in cases of prior hysterectomy, with or without coexisting stress urinary incontinence. All patients provided written informed consent before undergoing the procedure.

In some cases, the selection of the surgical approach was influenced by additional factors. These included the severity of prolapse, the patient's general health status and body weight, the urgency of the intervention, and the availability of the robotic system. In particular, patients with higher body mass index (BMI) scheduled for multiple procedures were preferentially assigned to robotic-assisted surgery, as recent studies have shown that elevated BMI is not a limiting factor for robotic techniques, unlike for conventional laparoscopy (12–15).

Due to shared access to the robotic system among three surgical departments, availability was limited to once every two weeks. The overall surgical goal was to restore apical support by elevating the cervix or, in post-hysterectomy patients, the vaginal vault.

### Surgical techniques

#### Laparoscopic sacrocolpopexy

Laparoscopic sacrocolpopexy was performed using a standardized, uniform technique. The optic trocar was placed 2 cm supraumbilically, and pneumoperitoneum was established via Veress needle following Semm's safety protocol. A preliminary inspection of the upper abdominal organs (stomach, liver, peritoneum, appendix) was conducted to confirm operative feasibility.

The patient was then positioned in Trendelenburg. Two additional trocars (10 mm left, 5 mm right) were inserted in the lower abdomen, along with a 5 mm left paraumbilical trocar. After insufflation, the pelvis and abdomen were examined with attention to ureteral anatomy. A VASA/CESA vaginal manipulator was inserted and operated by the resident to aid intraoperative exposure.

In patients without prior hysterectomy, supracervical hysterectomy was performed. A folded Y-shaped DynaMesh® PVDF mesh was tunneled along the right sacrouterine ligament and used to suspend the cervix or vaginal vault to the sacral promontory. The mesh was anchored to the anterior and posterior aspects of the cervix or vault using eight individual button sutures (four ventral, four dorsal) placed with non-resorbable PremiCron® thread. Button sutures refer to interrupted, individually tied sutures that allow for secure, tension-adjusted fixation.

Two additional sutures reinforced the attachment at the cervix or vault scar. The distal end of the mesh was fixed to the promontory using two button sutures or staples. Mesh coverage was achieved through peritonealization using resorbable V-Loc™ sutures, reapproximating the bladder and posterior peritoneum. All knots were tied intracorporeally.

#### Robotic-assisted sacrocolpopexy

In patients with obesity, the procedure began by taping the abdominal pannus cranially to prevent interference and facilitate optimal trocar placement. The patient was then positioned in Trendelenburg before docking the robotic system.

Robotic-assisted sacrocolpopexy was performed using a modified port placement technique. After positioning, the Da Vinci® Surgical System (Intuitive Surgical, Inc., Sunnyvale, CA) was docked to three operative ports and a camera port, all placed in a horizontal line approximately 2 cm supraumbilically. An additional assistant trocar was inserted in the lower right quadrant, positioned 2 cm caudal to the main trocar line. This layout allowed clear visualization and helped reduce the risk of trocar-related complications.

All subsequent procedural steps—including dissection, mesh placement, fixation to the cervix or vaginal vault and promontory, and peritonealization—were performed identically to the laparoscopic approach, using the same mesh material, suture types, and anatomical landmarks.

### Outcomes and data collection

The primary outcomes of this study were duration of surgery and length of postoperative hospital stay. Surgery duration was defined as the time from the initial skin incision to final suture, as recorded in the operating room protocol. To enhance comparability, durations of concomitant procedures were estimated and subtracted using historical averages: approximately 20 min for mid-urethral sling placement and 20 min each for anterior or posterior colporrhaphy (10).

Length of stay was calculated from the day of surgery to the day of discharge, excluding any preoperative admission days to avoid bias. These outcomes were analyzed in relation to age, BMI, and parity to assess their impact on surgical efficiency and recovery.

Secondary outcomes included intraoperative complications (e.g., cystotomy, colotomy, vaginotomy, anesthesia-related events) and postoperative complications (e.g., wound infections, urinary tract infections, cardiovascular incidents, or other medical conditions requiring intervention or extended care). All complications were extracted from patient records and classified as either directly surgery-related or general postoperative events. Patient demographics and clinical data were retrieved from a dedicated institutional database. Variables included: age, BMI, parity and delivery mode (vaginal or cesarean), prior pelvic surgery, surgical method (LSC or RASC), surgical duration, hospital stay, and complications.

BMI was classified according to international standards: BMI 25.0–29.9 kg/m^2^ as overweight and ≥30.0 kg/m^2^ as obese (16). All patients underwent a standardized preoperative assessment, including clinical examination, perineal ultrasound, urodynamic testing, and post-void residual measurement. They also completed the validated International Consultation on Incontinence Questionnaire (ICIQ) to evaluate symptom impact and report obstetric and surgical history.

Postoperative follow-up included perineal ultrasound on day 2 and at six months. Surgical success was defined as POP-Q stage 0 combined with complete resolution of prolapse-related symptoms.

### Statistical analysis

Continuous variables were expressed as mean values ± standard deviations (SD), while categorical data were presented as absolute frequencies and percentages. Differences in each variable between the two treatment groups (LSC vs. RASC) were summarized using descriptive statistics. Linear and logistic regression models were employed, depending on the nature of the outcome variables—linear regression for continuous endpoints and logistic regression for binary outcomes. The choice of these models was guided by the distribution and type of each endpoint, ensuring appropriate statistical handling of the data. Adjustment variables were selected based on clinical relevance and driven by domain knowledge and the underlying clinical questions. This approach aligns with the exploratory nature of the study and aims to provide meaningful insights while acknowledging the retrospective design.

A *p*-value of <0.05 was considered statistically significant. Given the exploratory nature of the study, no correction for multiple testing was applied. Consequently, the analyses should be interpreted as exploratory rather than confirmatory, in accordance with ICH E9 guidelines (17). All statistical analyses were conducted using the statistical software R, Version 4.3.3 (18) and the integrated development environment (IDE) RStudio, Version 2023.12.1.402 (19).

## Results

### Patient characteristics

A total of 162 women underwent minimally invasive sacrocolpopexy and were included in the final analysis: 104 (64%) via laparoscopic-assisted sacrocolpopexy (LSC) and 58 (36%) via robotic-assisted sacrocolpopexy (RASC), resulting in an approximate 2:1 group distribution (Table 1). Among them, 32 women (20%) were obese (BMI ≥30 kg/m^2^) and 66 (41%) were overweight (BMI 25–29.9 kg/m^2^). Approximately 35% (57/162) had previously undergone a hysterectomy, while two declined hysterectomy during the current procedure. Of these 57 patients, 17 (29%) were operated robotically and 40 (70%) by laparoscopic surgery, again resulting in a 2:1 group distribution (*p* = 0.318).

**Table 1:** Baseline patient characteristics in LSC vs. RASC groups.

Characteristic	LSC (*n* = 104)	RASC (*n* = 58)	Total (*n* = 162)	*p*-value
Age [years], mean (SD)	63.82 (±11.67)	64.38 (±10.53)	64 (±11.2), Range 32–87	0.761
BMI [kg/m^2^], mean (SD)	26.36 (±4.38)	27.24 (±4.98)	26.7 (±4.6), Range 18–42	0.610
• Normal weight, *n* (%) (BMI < 25 kg/m^2^),	44 (42.3%)	20 (34.5%)	64 (40%)	0.418
• Overweight, *n* (%) (BMI 25.0–29.9 kg/m^2^)	40 (38.5%)	26 (44.8%)	66 (41%)	0.533
• Obese, *n* (%) (BMI ≥ 30 kg/m^2^)	20 (19.2%)	12 (20.7%)	32 (20%)	0.986
Parity, mean (SD)	2.28 (±1.1)	2.24 (±1.34)	–	0.848
• Vaginal births, mean (SD)	2.17 (±1.17)	2.14 (±1.28)	–	0.859
Previous Hysterectomy, *n* (%)	40 (38.5)	17 (29.3)	57 (35.1)	0.318

Demographic and clinical baseline characteristics of patients undergoing LSC or RASC. Values are presented as mean ± standard deviation (SD) or counts with percentages, as appropriate.

BMI, body mass index; LSC, laparoscopic sacrocolpopexy; RASC, robotic-assisted sacrocolpopexy; SD, standard deviation.

Regarding the 19 patients (12%) with previous POP surgery, 9 patients (12%) had a re-surgery via RASC and 10 patients (12%) via LSC.

All of the 58 patients with robotic assisted- and all of the 104 laparoscopic operated sacrocolpopexy achieved POP-Q Stage 0 postoperatively, and no conversion to open surgery was required. Additional vaginal procedures were performed in some cases and are detailed in the outcomes section.

Baseline characteristics such as age, parity, and number of vaginal births were comparable between the two groups (Table 1).

### Operation duration

The average uncorrected surgery duration was 188 ± 54 min for the LSC group and 199 ± 51 min for the RASC group. After correcting for concomitant procedures, the mean operative times were 180 ± 53 min (LSC) and 194 ± 49 min (RASC). Although RASC procedures tended to be longer, the difference between the groups was not statistically significant (*p* = 0.114) (Table 2).

**Table 2:** Perioperative outcomes and influencing factors in LSC vs. RASC.

Parameter	LSC (*n* = 104)	RASC (*n* = 58)	*p*-value	95%-CI
Surgery duration [min], mean (SD)	187.75 (±54.40)	198.72 (±50.7)	0.209	
• Corrected surgery duration[min], mean (SD)	180.06 (±53.20)	193.55 (±49.04)	0.114	
• Change per Unit BMI [min]	+2.8*	+2.0	LSC: 0.0177	0.50–5.14
			RASC: 0.1167	–0.53 to 4.63
• Change per childbirth [min]	+9.8*	+8.7	LSC: 0.0379	0.56–19.14
			RASC: 0.0698	0.50–5.14
Vaginal birth [min]	+7.7	+9.9	LSC: 0.0852	–1.09 to 16.52
			RASC: 0.050	–0.0017 to 19.8643
Cesarian section [min]	+15.8	−3.2	LSC: 0.326	–15.97 to 47.57
			RASC: 0.863	–39.63 to 33.31
• Change per year of age [min]	+0.4	+0.2	LSC: 0.364	–0.48 to 1.30
			RASC: 0.719	–1.02 to 1.47
Mean postoperative days (SD)	4.0 (±1.55)	4.72 (±4.01)	0.104	
• Age – increase in stay [days/year]	+0.031*	+0.001	LSC: 0.0152	0.006–0.057
			RASC: 0.876	−0.0213 to 0.0249
• Surgery duration – stay [days/minute]	+0.009*	−0.002	LSC: 0.0006	0.0042–0.0151
			RASC: 0.277	−0.0077 to 0.0023
• BMI – stay [days/unit]	+0.086*	+0.011	LSC: 0.0130	0.019–0.154
			RASC: 0.667	−0.038 to 0.060
• Births – stay [days/birth]	+0.304*	+0.270*	LSC: 0.0277	0.03–0.57
			RASC: 0.0026	0.10–0.44
Vaginal births – stay [days/birth]	+0.333*	+0.283*	LSC: 0.01	0.08–0.59
			RASC: 0.0030	0.10–0.47
Cesarian section – stay [days/birth]	−0.725	+0.236	LSC: 0.121	−1.64 to 0.20
			RASC: 0.483	−0.43 to 0.90

Comparison of surgical duration, length of hospital stay, and associated influencing factors between laparoscopic (LSC) and robotic-assisted (RASC) sacrocolpopexy groups. Regression analyses were used to assess the effect of age, BMI, parity, surgical year, and delivery mode on perioperative outcomes. Values are reported as means ± standard deviation or regression coefficients with corresponding *p*-values.

BMI, body mass index; LSC, laparoscopic sacrocolpopexy; min, minute; RASC, robotic-assisted sacrocolpopexy; SD, standard deviation, *n*, sample size.

*Statistically significant.

#### Influence of patient and procedural factors

##### Body mass index (BMI)

**Figure 1:**
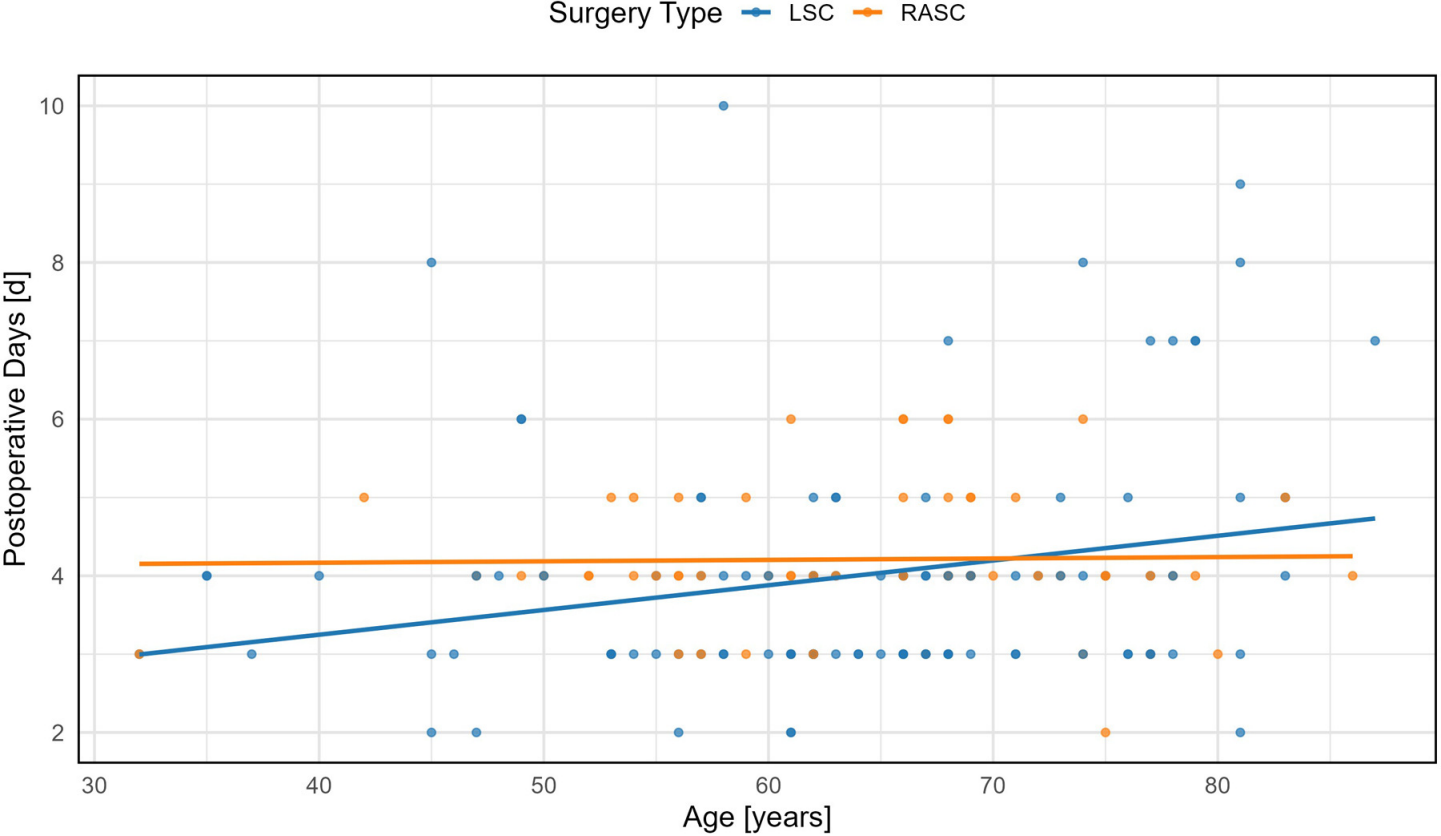
Association between patient age and postoperative length of stay. Linear regression analysis showing the relationship between age and postoperative hospital stay (in days) in women undergoing minimally invasive sacrocolpopexy. A statistically significant positive correlation was observed in the LSC group (*p* = 0.0152), indicating increased hospital stay with advancing age. No significant correlation was found in the RASC group.

A significant association was found between BMI and surgical duration in the LSC group (+2.82 min per BMI unit, *β* = 2.82, 95% CI: 0.50–5.14, *p* = 0.0177). No significant association was observed in the RASC group (+2.00 min per BMI unit, *β* = 2.05, 95% CI: −0.53–4.63, *p* = 0.1167) (Table 2 and Figure 1).

##### Parity

The number of births significantly prolonged surgical time in the LSC group (+9.8 min per birth, *β* = 9.85, 95% CI: 0.56–19.14, *p* = 0.0379). In the RASC group, the effect was similar in magnitude (+8.7 min per birth) but not statistically significant (*β* = 8.76, 95% CI: 0.50–5.14, *p* = 0.0698) (Table 2).

When stratified by delivery mode: Vaginal births were associated with time increases of +7.72 min (LSC: *β* = 7.72, 95% CI: −1.09–16.52, *p* = 0.0852) and +9.93 min (RASC: *β* = 9.93, 95% CI: −0.0017–19.8643, *p* = 0.050). These findings followed the overall trend but were not statistically significant. Cesarean sections also led to a delay in the LSC group (+15.80 min, *β* = 15.80, 95% CI: −15.97–47.57), while in the RASC group, surgeries were on average 3.16 min shorter in women with previous cesarean delivery (*β* = –3.16, 95% CI: −39.63–33.31). Statistical testing was limited due to small sample size (*n* = 14), so significance could not be reliably calculated (Table 2).

##### Age

Surgical duration increased slightly with advancing age, with a larger effect observed in LSC (+0.41 min per year, *β* = 0.41, 95% CI: −0.48–1.30, *p* = 0.364) compared to RASC (+0.22 min per year, *β* = 0.22, 95% CI: −1.02–1.47, *p* = 0.719). However, these associations did not reach statistical significance (Table 2).

##### Concomitant procedures

Additional vaginal surgeries were more common in the LSC group and likely contributed to longer operative times. Specifically, TVT placement occurred in 25.0% of LSC cases vs. 15.5% in RASC, and anterior colporrhaphy was performed in 10.6% of LSC cases vs. 3.4% in RASC. Posterior colporrhaphy occurred infrequently in both groups (three LSC vs. four RASC). While no formal comparison was made, the lower frequency of adjunct procedures in RASC supports shorter corrected times for comparable cases.

### Postoperative recovery

The mean number of postoperative stay was 4.0 ± 1.55 days in the LSC group and 4.72 ± 4.01 days in the RASC group. While the average hospital stay was longer for RASC, this difference was not statistically significant (Table 2).

#### Influence of patient and procedural factors

##### Age

**Figure 2:**
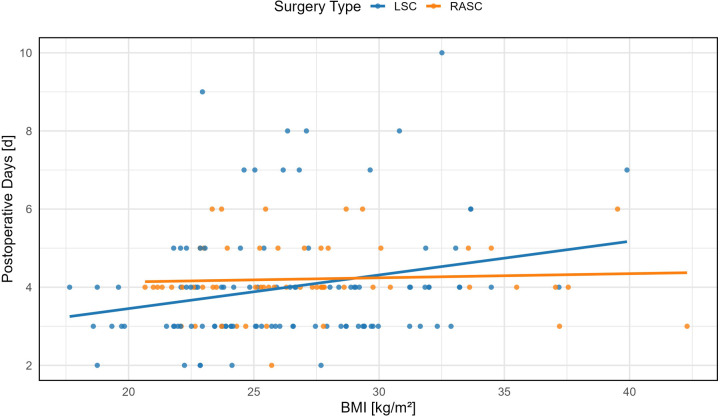
Correlation between postoperative length of stay and body mass index (BMI). Scatter plot depicting the association between BMI and postoperative hospital stay (in days) following minimally invasive sacrocolpopexy. A statistically significant positive correlation was observed in the LSC group (*p* = 0.0130), whereas no significant relationship was found in the RASC group.

In the LSC group, postoperative stay increased significantly with age (*β* = 0.031, 95% CI: 0.006–0.057, *p* = 0.015), while in the RASC group, the effect was negligible (*β* = 0.0018, 95% CI: −0.0213–0.0249, *p* = 0.876). For each additional year of life, LSC patients stayed 0.031 days longer, compared to 0.001 days in RASC (Table 2 and Figure 2).

##### Surgery duration

In LSC, a longer surgery significantly predicted a longer hospital stay (*β* = 0.0097*,* 95% CI: 0.0042–0.0151, *p* = 0.0006). For each additional operative minute, the postoperative stay increased by 0.009 days. In contrast, RASC showed no such effect (–0.002 days per minute; *β* = 0.011, 95% CI: −0.038 to 0.060, *p* = 0.667), indicating greater procedural tolerance despite longer mean operative times (Table 2).

##### BMI

**Figure 3:**
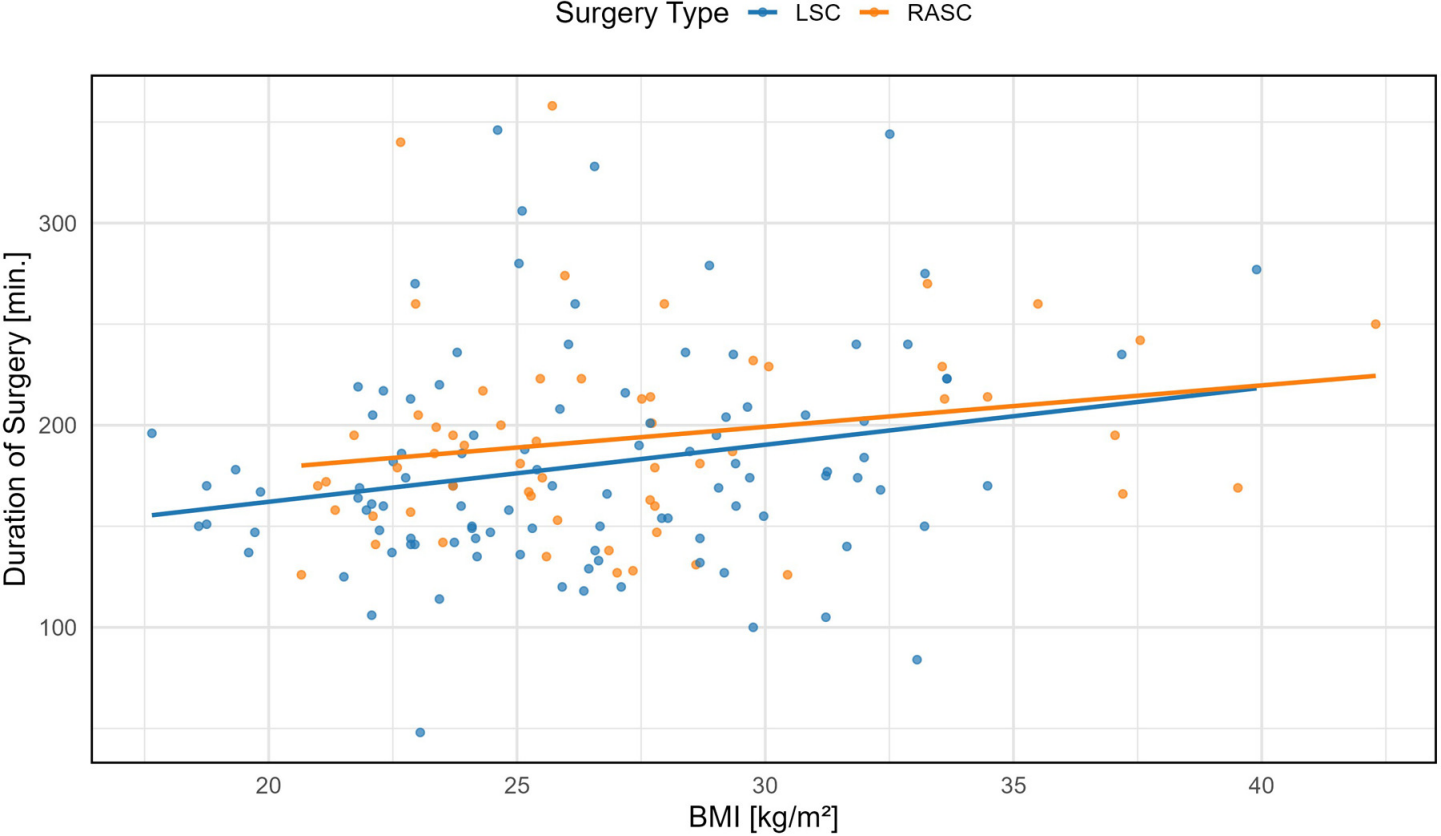
Association between body mass index (BMI) and corrected time of surgery in laparoscopic (LSC) and robotic-assisted (RASC) sacrocolpopexy. Linear regression lines with 95% confidence intervals are shown for each group. In the LSC group, a significant positive association was observed (LSC = + 2.82 min/unit BMI, 95% CI: 0.50–5.14, *p* = 0.0177), whereas the trend in the RASC group was not statistically significant (RASC = + 2.00 min/unit BMI, 95% CI: −0.53 to 4.63, *p* = 0.1167).

BMI significantly affected hospital stay in the LSC group (*β* = 0.086, 95% CI: 0.019–0.154, *p* = 0.013), where each unit increase led to an average of +0.09 days. In RASC, the corresponding increase was only +0.01 days, and not statistically significant (*β* = 0.011, 95% CI: −0.038 to 0.060, *p* = 0.667) (Table 2 and Figure 3).

##### Year of surgery

Earlier surgical years were associated with longer stays in LSC patients. Conversely, in RASC, later years were associated with increased postoperative stays. However, the implementation timeline also varied, with LSC performed from 2010 on and RASC introduced in 2016, creating temporal imbalances. Over the years, the length of postoperative hospital stay significantly decreased for LSC procedures [−0.17 days per year (95% CI: −0.25, −0.08)] but increased for RASC procedures [+0.25 days per year (95% CI: 0.13, 0.37)]. When considering all procedures, there was an average annual reduction of 0.10 days [95% CI: −0.17, −0.03]. Notably, RASC, introduced six years after LSC, was already associated with a 0.6-day shorter postoperative stay at baseline.

##### Parity

The number of previous births influenced hospital stay in both groups. In LSC, each additional birth increased postoperative days by 0.304 days (*β* = 0.30, 95% CI: 0.03–0.57, *p* = 0.0277), compared to 0.270 days in RASC (*β* = 0.27, 95% CI: 0.10–0.44, *p* = 0.0026). When separated by birth mode: Vaginal births increased stay by 0.333 days in LSC (*β* = 0.33, 95% CI: 0.08–0.59, *p* = 0.01) and 0.283 days in RASC (*β* = 0.28, 95% CI: 0.10–0.47, *p* = 0.0030). Cesarean sections had opposite effects: hospital stay decreased in LSC by −0.72 days (*β* = −0.72, 95% CI: −1.64–0.20), while it increased in RASC by +0.24 days (*β* = 0.24, 95% CI: −0.43–0.90), not statistically tested due to small sample size (Table 2).

To further explore factors associated with longer hospital stays, a binary classification was applied, dividing patients into two groups: those who stayed less than six days and those who stayed six days or more after surgery. Although only 12% of patients fell into the longer-stay group (≥6 days), this subgroup consistently exhibited a cluster of high-risk characteristics. These included a significantly older age (69.1 ± 11.7 vs. 63.3 ± 11.0 years, *p* = 0.032), higher BMI (29.1 ± 5.0 vs. 26.3 ± 4.5 kg/m^2^, *p* = 0.011), and a greater number of previous births (2.80 ± 1.0 vs. 2.19 ± 1.2, *p* = 0.031).

Additionally, patients in this group had a markedly longer surgery duration (222.6 ± 63 vs. 179.6 ± 48 min), and a significantly higher incidence of intra- and postoperative complications (intraoperative: 7 (35.0%) vs. 4 (2.8%), postoperative: 9 (45.0%) vs. 13 (9.2%), *p* < 0.001). These findings suggest that prolonged hospitalization was associated with a combination of older age, higher body weight, surgical complexity, and the occurrence of postoperative adverse events.

### Complication profiles

Of the 162 minimally invasive sacrocolpopexy procedures, 133 (82%) were complication-free. Intraoperative complications occurred in 11 cases (7%), and postoperative complications in 22 cases (14%), resulting in an overall complication rate of 18% since four patients showed intraoperative as well as postoperative complications and were counted as one case (Table 3). Notably, no procedures required conversion to open surgery. Intraoperative events included cystotomy, colotomy, vaginotomy, and subcutaneous emphysema related to CO₂ insufflation. Postoperative complications involved hematomas, urinary tract infections (UTIs), and persistent emphysema, as well as other adverse events requiring intervention or prolonging hospital stay. To improve statistical power, intra- and postoperative complications were analyzed jointly, given a significant correlation between them (*p* = 0.0278). All data were extracted from operative and medical records.

**Table 3:** Intraoperative and postoperative complications in LSC and RASC groups.

Complication type	Subtype	LSC (*n* = 104)	RASC (*n* = 58)	Total (*n* = 162)	*p*-value
Intraoperative	Cystotomy	5		5	
	Colotomy^b^		2	2	
	Vaginotomy	1		1	
	Cardiovascular (hypertensive crisis)	2		2	
	Skin reaction (swelling, emphysema)		1	1	
	Subtotal	8 (7.7%)	3 (5.2%)	11 (6.8%)	0.7476
Postoperative	Urinary tract infection	1	2^a^	3	
	Cardiovascular	4	3	7	
	Respiratory	1	1	2	
	Hematologic	4	1	5	
	Immune-related	2	1	3	
	Skin reaction		1	1	
	Pain		1	1	
	Subtotal	12 (11.5%)	10 (17.2%)	22 (13.6%)	0.3435
Overall		18^c^ (17.3%)	11^c^ (19%)	29^c^ (17.9%)	0.8323

Patients with intraoperative and postoperative complications in LSC and RASC groups.

Overview of complication rates observed during and after laparoscopic (LSC) and robotic-assisted (RASC) sacrocolpopexy per patient. Complications include intraoperative events (e.g., cystotomy, colotomy) and postoperative issues (e.g., urinary tract infections, hematoma). Overall complication rates, as well as distribution by surgical approach and fixation type, are presented.

This table serves to illustrate the distribution and frequency of complications.

LSC, laparoscopic sacrocolpopexy; RASC, robotic-assisted sacrocolpopexy; UTI, urinary tract infection.

^a^ This patient in the RASC group had both a UTI and an immune related complication postoperatively.

^b^ Colotomy: an unintended surgical incision into the colon, typically resulting from dissection or mesh placement complications.

^c^ 4 Patients showed intraoperative as well as postoperative complications.

Higher BMI was associated with complications in the LSC group (28.4 for intraoperative, 26.8 for postoperative). In contrast, RASC patients with higher BMI showed fewer complications, possibly reflecting a learning curve or refined patient selection. Additionally, mesh fixation to the vaginal vault was linked to a higher complication rate compared to cervical fixation, indicating the anatomical site may influence surgical risk; 68 patients had a fixation at the vaginal vault. Of the 29 patients with complications, 19 patients (65.5%, *p* *=* *0.011*) had a fixation of the vaginal vault with the following complications: 5 patients with a cystotomy and 2 with a colotomy, 4 patients with high blood pressure, 2 with hematological issues and 2 with respiratory issue, as well as others complications concerning just one patient at a time.

## Discussion

The aim of this study was to evaluate whether robotic-assisted sacrocolpopexy (RASC) offers measurable advantages over laparoscopic sacrocolpopexy (LSC) in relation to patient age, body mass index (BMI), and parity, with a particular focus on surgical duration, length of hospital stay, and complication rates. Our study contributes to the growing body of evidence suggesting that both LSC and RASC are safe and effective procedures for the treatment of pelvic organ prolapse (POP), but they diverge in their sensitivity to patient-specific factors.

In LSC, surgery duration and length of hospital stay were significantly influenced by higher BMI, advanced age, and greater parity. In contrast, these variables had minimal impact on RASC outcomes. Complication rates were similar between groups overall, though a trend toward fewer complications in obese patients undergoing RASC was observed. Notably, the majority of intraoperative organ injuries occurred in the LSC group, and fixation at the vaginal vault—rather than the cervix—was associated with a higher complication risk.

### Discussion of the results in the context of the scientific literature

#### Surgical duration

While robotic-assisted procedures are often assumed to take longer due to docking and setup times, our data revealed no statistically significant difference in corrected surgical duration between LSC and RASC. This observation aligns with findings from Tan-Kim et al. and Chang et al., who also reported comparable or slightly shorter operative times in robotic sacrocolpopexy compared to laparoscopy (10, 20, 21).

However, our subgroup analysis showed that surgery duration increased significantly with BMI and parity in the LSC group, while remaining relatively stable in RASC. This suggests that RASC may be less sensitive to patient-related complexity. Similar trends have been observed in other gynecologic and urologic surgeries, where robotic systems demonstrated greater performance consistency in obese and high-risk patients (12, 13, 22, 23).

Regarding age, literature on its impact is mixed. While our data suggest increasing age prolongs laparoscopic sacrocolpopexy, a study by Moreno-Mira et al. (2015) reported longer operative times in younger patients, possibly due to anatomical differences, more complex procedural demands, or surgeon caution in fertility-preserving contexts (24). These divergent findings underscore that age-related effects may depend heavily on surgical setting, case selection, and technique.

With regard to evaluating the specific impact of parity on surgical duration in sacrocolpopexy, we were unable to identify any published data.

#### Postoperative recovery

Although the overall length of postoperative hospital stay did not differ significantly between RASC and LSC in our cohort, our subgroup analysis revealed that this parameter was more strongly influenced by patient characteristics in the LSC group. Specifically, age, BMI, and number of births were each significantly associated with longer postoperative stays after LSC, whereas these variables had minimal or no impact in the RASC group. This suggests that robotic-assisted surgery may provide a more stable postoperative course for anatomically or physiologically complex patients.

Our findings partially contrast with existing literature. Multiple studies, including those by Menzella et al. (2013) and Mahoney et al. (2019), reported no significant difference in length of hospital stay between LSC and RASC, regardless of body weight (25, 26). Similarly, Chang et al. also found no difference in hospital stay between the two surgical modalities (20). The discrepancy may stem from differences in study design, institutional discharge protocols, or patient selection strategies.

As for age, our data showed that older patients tended to remain hospitalized longer after LSC. However, this was not confirmed by another study, which reported no significant differences in length of hospital stay across different age groups (24). This discrepancy could be due to differences in surgical procedures, comorbidity profiles, or discharge criteria, and highlights the need for more targeted research on the influence of age in minimally invasive POP surgery.

We were unable to identify published studies that directly address the impact of parity on length of hospital stay in the context of sacrocolpopexy.

#### Complications

In our cohort, overall complication rates were low (18%), with no statistically significant difference between LSC and RASC. This is consistent with previous randomized trials and meta-analyses comparing minimally invasive approaches for POP repair, which have found comparable safety profiles between laparoscopic and robotic sacrocolpopexy (9, 27).

However, when stratified by BMI, we observed a trend toward fewer complications in obese patients undergoing RASC, whereas complication rates in obese LSC patients were slightly elevated. This contrasts with recent studies which reported no significant interaction between BMI and complication rates for either surgical approach (25). This suggests that while robotic surgery may offer technical advantages in high-BMI patients, current evidence is insufficient to confirm a protective effect based on BMI alone.

Notably, we recorded six intraoperative organ injuries in the LSC group, compared with only two in the RASC group. This supports the hypothesis that robotic systems may offer improved visualization and stability, especially in complex pelvic dissections. These findings align with literature emphasizing the advantages of robotic platforms in providing precise access to deep pelvic landmarks, particularly in anatomically challenging patients (23).

In our study, complications were more frequent when mesh was fixed to the vaginal vault rather than the cervix. While direct comparative data are limited, prior work by Visco et al. (2001) showed that vaginal mesh placement or suture passage—as used in combined vaginal–abdominal colpoperineopexy—was associated with significantly higher mesh erosion rates and shorter time to erosion (28). Even though Tius et al. (2025) did not distinguish between fixation at the cervix vs. the vaginal vault but rather focused on uterus-preserving vs. hysterectomy-combined surgery, they showed that no significant complications were related to the hysterectomy itself when combined with sacrocolpopexy (29).

This indicates that both the surgical approach and fixation site may influence complication rates. However, these findings should not be generalized to abdominal-only approaches, and further research is needed to determine whether fixation to the vaginal vault independently increases complication risk compared to cervical fixation in minimally invasive sacrocolpopexy.

Finally, the lower rate of concomitant vaginal procedures (e.g., anterior/posterior colporrhaphy, TVT placement) in the RASC group may reflect more robust apical support achieved with robotic dissection and mesh fixation. Improved anatomical restoration with robotic assistance may reduce the need for adjunctive repairs. This observation is clinically relevant and warrants further investigation into whether robotic sacrocolpopexy reduces the need for additional vaginal compartment interventions.

### Clinical implications

The clinical implications of these findings are relevant for surgical planning and healthcare resource allocation. Although RASC is associated with higher equipment and maintenance costs (27, 30, 31), our study suggests that these may be offset by reduced sensitivity to patient complexity and potentially shorter recovery times in high-risk populations. This is especially pertinent given rising global obesity rates and the growing demand for POP surgery (32, 33). Additionally, RASC patients in our cohort required fewer concomitant procedures such as TVT or anterior/posterior colporrhaphy, which may reflect improved pelvic floor restoration through robotic precision.

The burden of pelvic floor disorders extends beyond the anatomical level and can significantly impact daily function and psychological well-being. While our cohort consisted of older women with advanced POP, pelvic floor dysfunction can also affect younger, athletic populations. For instance, Rubin et al. (2023) found that urinary incontinence (UI) was present in 78% of high-performance swimmers and was significantly associated with reduced quality of life, even in the absence of prolapse (34). Although UI and POP are distinct conditions, they often share underlying pathophysiology and risk factors such as pelvic floor muscle weakening, parity, and high-impact physical strain. These findings underscore the importance of early recognition and effective, tailored interventions across the spectrum of pelvic floor dysfunction, particularly in symptomatic cases requiring surgical repair.

Management of pelvic floor disorders, including POP and lower urinary tract dysfunctions, increasingly relies on tailored approaches based on symptom severity, patient characteristics, and response to prior treatments. While our study focused on anatomical restoration through sacrocolpopexy, other modalities like sacral neuromodulation (SNM) offer therapeutic options for patients with refractory urinary symptoms such as urgency, frequency, or retention. In a retrospective cohort study, Culmone et al. (2022) demonstrated that SNM significantly reduced post-void residual volumes, leak episodes, and voiding frequency in patients with treatment-resistant bladder dysfunctions (35). These findings underscore the value of individualized surgical and non-surgical interventions across the spectrum of urogynecological conditions, particularly when first-line treatments prove insufficient.

However, not all institutions may have access to robotic systems due to cost constraints. Our findings do not suggest that RASC should replace LSC universally, but rather that it should be considered a valuable complement, especially for selected subgroups such as obese patients, elderly women, or those with high parity. Hospitals considering the purchase of a surgical robot should evaluate the cost–benefit ratio considering their patient population and case complexity.

### Study limitations and future directions

This retrospective, non-randomized study is subject to selection bias, as the choice of LSC or RASC was based on clinical and logistical factors. The implementation timeline also varied, with LSC performed from 2010 and RASC introduced in 2016, creating temporal imbalances. Limited access to the robotic system contributed to the uneven group sizes. Additionally, length of hospital stay is partially influenced by institutional and reimbursement policies. While Germany's DRG system does not mandate specific durations, our hospital's internal protocol sets a minimum stay of 3–5 days after pelvic floor surgery, which may limit international generalizability. However, the use of a single, experienced surgeon throughout minimizes operator bias. Future prospective studies should further evaluate RASC in high-risk subgroups and assess its cost-effectiveness relative to patient complexity.

In conclusion, this study shows that both laparoscopic and robotic-assisted sacrocolpopexy are safe and effective for POP repair, but robotic-assisted surgery demonstrates greater procedural stability and efficiency in patients with higher BMI, older age, and greater parity. These patient factors were associated with longer surgery duration and hospital stay in the laparoscopic group. While complication rates were similar overall, intraoperative injuries were more frequent in LSC. Given its reduced sensitivity to anatomical and physiological complexity, robotic sacrocolpopexy may provide a strategic clinical advantage in challenging cases, highlighting the importance of individualized, evidence-based surgical planning that accounts for patient-specific risk factors and comorbidities. This is particularly relevant given the aging population and the global rise in obesity rates, both of which are contributing to an increasing number of anatomically complex surgical cases.

## Data Availability

The raw data supporting the conclusions of this article will be made available by the authors, without undue reservation.
